# Developmental reprogramming of myometrial stem cells by endocrine disruptor linking to risk of uterine fibroids

**DOI:** 10.1007/s00018-023-04919-0

**Published:** 2023-08-31

**Authors:** Qiwei Yang, Mohamed Ali, Lindsey S. Treviño, Aymara Mas, Ayman Al-Hendy

**Affiliations:** 1 Department of Obstetrics and Gynecology, University of Chicago, 5841 S. Maryland Ave., Chicago, IL 60637 USA; 2 Division of Health Equities, Department of Population Sciences, City of Hope, Duarte, CA 91010 USA; 3 Center for Precision Environmental Health and Department of Molecular and Cellular Biology, Baylor College of Medicine, Houston, TX 77030 USA; 4 Carlos Simon Foundation, INCLIVA Health Research Institute, Avda. Menéndez Pelayo 4, 46010 Valencia, Spain

**Keywords:** Leiomyoma, Progenitor cells, Eker rat, Developmental reprogramming, Endocrine-disrupting chemicals, Estrogen signaling, Hyper-estrogenic, Estrogen-responsive genes, Hormonal imprint, Epigenome, MLL1 activation, TASP1, DNA hypo-methylation

## Abstract

**Background:**

The stage, when tissues and organs are growing, is very vulnerable to environmental influences, but it’s not clear how exposure during this time causes changes to the epigenome and increases the risk of hormone-related illnesses like uterine fibroids (UFs).

**Methods:**

Developmental reprogramming of myometrial stem cells (MMSCs), the putative origin from which UFs originate, was investigated in vitro and in the Eker rat model by RNA-seq, ChIP-seq, RRBS, gain/loss of function analysis, and luciferase activity assays.

**Results:**

When exposed to the endocrine-disrupting chemical (EDC) diethylstilbestrol during Eker rat development, MMSCs undergo a reprogramming of their estrogen-responsive transcriptome. The reprogrammed genes in MMSCs are known as estrogen-responsive genes (ERGs) and are activated by mixed lineage leukemia protein-1 (MLL1) and DNA hypo-methylation mechanisms. Additionally, we observed a notable elevation in the expression of ERGs in MMSCs from Eker rats exposed to natural steroids after developmental exposure to EDC, thereby augmenting estrogen activity.

**Conclusion:**

Our studies identify epigenetic mechanisms of MLL1/DNA hypo-methylation-mediated MMSC reprogramming. EDC exposure epigenetically targets MMSCs and leads to persistent changes in the expression of a subset of ERGs, imparting a hormonal imprint on the ERGs, resulting in a “hyper-estrogenic” phenotype, and increasing the hormone-dependent risk of UFs.

**Supplementary Information:**

The online version contains supplementary material available at 10.1007/s00018-023-04919-0.

## Introduction

Uterine fibroids (UFs) are the most common benign tumors of the reproductive tract and the most common indication for hysterectomy [1–3]. By the age of 50, up to 75% of all women develop at least one fibroid, and 15–30% of these women will develop severe symptoms associated with the presence of these tumors. Despite their high prevalence, the exact pathogenesis of these tumors is still largely unknown [4, 5].

Many risk factors have been recognized as contributors to the development of UF, including exposure to unfavorable environmental conditions [5]. Specifically, there is compelling evidence that exposure to hormones during development may be linked to a predisposition of the myometrium to UF development [6, 7]. Developmental exposure to endocrine-disrupting chemicals (EDCs), such as diethylstilbestrol (DES), phthalates, and the soy phytoestrogen genistein [8–11], has been shown to increase the incidence, multiplicity, and overall size of UFs in animal models. Concomitantly, epidemiologic studies have found an association between increased risk for early UF diagnosis and EDC exposure, as well as the use of soy-based formula during infancy [11–14].

Considering that UFs are monoclonal tumors, it is possible that the dysregulation of committed cells that acquire stem-like features could be responsible for this benign condition [15]. An increasing body of evidence supports the hypothesis that UFs originate from stem cells (SC) in the myometrium. Our group and others have conducted several studies to identify UF-initiating cells (UFICs) in UFs [16–19]. Notably, one of the main differences between myometrial stem cells (MMSCs) and UFICs is at the genetic level, since specific mutations in *MED12* observed in UFICs, are not present in MMSCs [19]. Due to the critical role of *MED12* in various cellular functions, the emergence of *MED12* mutations may be considered as one of the key factors involved in the conversion of MMSCs to UFICs. Given the fact that the DNA repair machinery is extensively involved in genetic mutation and genome instability in several diseases [20–23], we recently characterized the relationship between DNA repair capacity and genetic stability in UFs [24]. Our studies demonstrated that several key DNA repair proteins are downregulated in UFs compared to myometrial tissues [23]. Moreover, rat MMSCs (rMMSCs) exposed to EDCs during early life exhibited decreased DNA repair capacity compared to normal human MMSCs and vehicle-exposed rMMSCs, respectively, highlighting the vital role of DNA damage repair pathways in UF pathogenesis [24–27].

With a full understanding of the mechanisms through which environmental EDCs affect the human epigenome, it can be easier to provide clear clinical guidance to address the potential health effects of low-level exposures that are commonly experienced within the general population. In addition, human studies that address environmental exposures are limited in feasibility by ethical concerns for human safety. Therefore, studies in animal models provide great opportunities to reveal links between early-life exposure to EDCs and related diseases [27]. Recently, our laboratory utilized Eker rats, which carry a germ-line mutation in the tuberous sclerosis complex 2 (*Tsc2*) tumor suppressor gene and are susceptible to developing UFs, to characterize potential gene-environment interactions. We isolated MMSCs from normal rat myometrium using Stro-1/CD44 surface markers. These Stro-1^+^ /CD44^+^ MMSCs also responded to environmental cues, contracting with age, and expanding in response to developmental exposures to EDCs that promote UF development [7]. Translating these findings to human, we found that the number of MMSCs in women myometrium correlated with the risk of developing UFs. Increased numbers of MMSCs were observed in Caucasian (CC) women with UFs as compared to CC women without UFs, and African American (AA) women, the group at the highest risk for these tumors, had the highest number of MMSCs [7]. Together, these findings provide the first direct evidence that Stro-1^+^ /CD44^+^ MMSCs as myometrial stem/progenitor cells are targets for ethnic and environmental factors contributing to the increased risk of UF development.

Although MMSCs have been identified as the cells from which UFs originate [19, 28–31], the epigenetic mechanism of MMSC programming due to developmental exposure to EDCs has not been characterized [6]. In the present study, we first assessed whether developmental exposure to EDCs reprograms MMSCs. We then determined how developmental EDC exposure triggers alterations in the epigenome. Our findings reveal that early-life exposure to EDC during sensitive periods of uterine development increases the risk of UF development by altering the activity of key epigenetic regulators, leading to modulation of the MMSC epigenome toward a pro-fibroid epigenomic landscape.

## Materials and methods

### Exposure of neonatal rats to the EDC

All experiments with female Eker rats (Long Evans; *Tsc-2*^Ek/+^) were approved by the Augusta University Animal Care Committee and Baylor College of Medicine. In all cases, the estrous stage of the animals was determined by histological examination of the vagina and analyzing the levels of ovarian hormones in their blood serum. To determine the impact of early-life environmental exposure on MMSCs, neonatal female Eker rats obtained from an on-site colony were subcutaneously injected with 10 µg of diethylstilbestrol (EDC, Sigma, St. Louis, MO, USA) per rat per day (*n* = 5) or with 50 µl of sesame seed oil (vehicle, VEH, *n* = 5) on days 10, 11, and 12 after birth (PND10-12) according to our previously described protocol [32]. The animals were maintained until they reached five months of age, at which time they were euthanized and subjected to myometrial Stro-1^+^/CD44^+^ stem cell isolation.

### Isolation and characterization of myometrial Stro-1^+^/CD44^+^ cells from Eker rats exposed to EDC and VEH

Myometrial Stro-1^+^/CD44^+^ cells from Eker rats were isolated as previously described [7]. Briefly, uterine tissues from Eker rats were collected and rinsed in wash buffer solution (Life Technologies, Grand Island, NY, USA). The myometrial layer was isolated by removing the endometrium and serosa with a sterile scalpel. Subsequently, freshly isolated myometrial cell suspensions were stained with antibodies to the cell surface proteins Stro-1 and CD44 (BD Biosciences, San Jose, CA, USA) and sorted by flow cytometry. Simultaneously, we also collected Stro1^−^/CD44^−^ cells from DES-exposed myometria. The isolated Stro-1^+^/CD44^+^ and Stro1^−^/CD44^−^ cells were grown in collagen-coated dishes in Smooth Muscle Growth Medium (SmGM, Lonza, Walkersville, MD, USA) at 37 °C in an incubator in a humidified atmosphere at 2% hypoxia. Characterization of rMMSCs was performed as previously described [7].

### Immunofluorescence and laser confocal microscopy

Immunofluorescence imaging of targeted proteins was performed as previously described [25, 33]. EDC-MMSCs and VEH-MMSCs were cultured on sterile glass coverslips in 6-well plates. The cells were fixed in a 4% formaldehyde solution at room temperature for 15 min. After washing in PBS 3 times, the cells were permeabilized for 15 min using 0.1% Triton X-100/PBS, and nonspecific binding was inhibited by incubating the cells for 1 h in blocking/incubation solution containing 1% BSA in 0.1% Triton X-100/PBS. Incubation with primary anti-H3K4me3 (1:200) antibody (Table S1) was performed for 2 h and was followed by incubation with anti-rabbit Alexa Fluor 555-conjugated secondary antibody (Thermo Fisher Scientific, Waltham, MA, USA) (1:1000) for 1 h, at room temperature. Finally, cells were washed for 15 min (three washes of 5 min each) with the above-described incubation solution, air-dried, and mounted on microscope slides with one drop of Fluorshield (Sigma) containing 4′,6-diamidino-2-phenylindole (DAPI) for nuclear staining. Fluorescent signals were visualized using a Zeiss 780 upright laser confocal fluorescence microscope and ZEN Black 2012 confocal software. Images were captured at 40 × magnification using a 40 × Plan-Apo (oil)—1.4 NA lens and exported using Zen Blue 2012 software. The experiments were conducted with biological replicates.

### Myometrial Stro-1^+^/CD44^+^ cell culture and treatment

Cell culture plates were treated with attachment factor protein (Cat# S-006-100, Thermo Fisher Scientific) for 5–10 min. Eker rat myometrial Stro-1/CD44^+^ cells were grown in SmBM medium supplemented with 5% fetal bovine serum, 0.1% insulin, 0.2% hFGF-B, 0.1% GA-10000, and 0.1% hEGF (Lonza). The cells were maintained at 37 °C in an incubator in a humidified atmosphere at 2% hypoxia.

To determine the expression of ERGs in response to estradiol treatment, EDC-MMSCs and VEH-MMSCs were grown in endothelial cell growth basal medium (phenol-free, CC-3129, Lonza) with 5% charcoal-stripped FBS (Thermo Fisher) for at least 3 days. First, the cells were plated in 6-well dishes. On the next day, 10 nM estradiol was added into the medium for 48 h. After treatment, the cells were collected for cDNA synthesis and qPCR analysis.

### Measurement of ERE-luciferase activity

The VEH-MMSCs and EDC-MMSCs were infected with Ad-ERE-luc overnight (16 h) (multiplicity of infection (MOI) = 10). The infection medium was then removed and replaced with the maintenance medium. 10 nM estradiol was added daily, and cells were treated for two days. Luciferase activities were determined using luciferase enzyme assay systems, according to the supplier’s protocol (Promega, Madison, WI). After completing the estradiol treatment, the lysates were prepared using Glo Lysis buffer. The luciferase activity was adjusted based on the cell count, which was determined by measuring the luminescence signal using the LDH-Glo cytotoxicity assay (Promega). Two independent experiments were carried out, with each experiment performed in triplicate.

### Whole transcriptome sequencing (RNA-seq) and quantitative real-time RT-PCR (RT-qPCR)

To identify developmentally reprogrammed EDC gene targets, whole-genome transcriptome sequencing and data analysis were performed using myometrial Stro-1^+^/CD44^+^ stem cells obtained from the uteri of 5-month-old animals that had been exposed to EDC on PND10-12. The RNeasy RNA isolation kit (Qiagen, Valencia, CA) was used to isolate RNA for RNA-seq analysis. RNA samples were treated with DNase I, and the purity and the quality of RNA were checked using a Bioanalyzer prior to cDNA synthesis. cDNA libraries were constructed using SPRI-works Fragment Library System I (Beckman Coulter, Brea, CA), and were then PCR-enriched and purified. For cluster generation, 10 pM cDNA was loaded into the paired-end flow cell on cBOT and then loaded on the HiSeq2500 platform (Illumina Inc., San Diego, CA, USA) to generate single 36 bp sequence reads. Sequence reads were aligned to the rat reference genome rn6 using Hisat2, and aligned read counts were analyzed using EdgeR for differential gene expression.

For qPCR, the same RNA used for RNA-seq was reverse-transcribed into first-strand cDNA with the Superscript III cDNA Transcription Kit (Invitrogen) using standard techniques. All assays were conducted in 96-well format, and each sample was run in triplicate. Real-time fluorescence detection of PCR products was performed using the following thermo-cycling conditions: 1 cycle of 95 °C for 2 min followed by 40 cycles of 95 °C for 5 s, and 60 °C for 30 s. The sequences of the primers used in this study are listed in Table S2. 18S ribosomal RNA was used as an endogenous control for gene expression. For data analysis, the comparative method (∆∆*C*_t_) was used to calculate the relative quantities of nucleic acid sequence.

### Chromatin immunoprecipitation (ChIP), ChIP-sequencing, and ChIP-qPCR

Chromatin immunoprecipitation sequencing (ChIP-seq) was performed as previously described [34, 35]. To obtain chromatin from Stro-1^+^/CD44^+^ MMSCs, the EZ-Magna ChIP™ kit (Millipore, Billerica, MA, USA) was employed following the instructions provided by the manufacturer. Cells were treated with 1.5% formaldehyde to crosslink proteins to DNA; then, they were incubated with 125 mM glycine and washed with chilled PBS. After centrifugation, cells were re-suspended in cell lysis buffer (PBS containing 0.5 mM EDTA and 0.05% Triton X-100) and collected via centrifugation. Each cell pellet was re-suspended in nuclear lysis buffer (50 mM Tris–HCl, pH 8.1, 10 mM EDTA, 1% SDS). Chromatin preparations were sonicated in a Bioruptor (Diagenode, Denville, NJ) to obtain fragments comprising 100–500 bp in length. ChIP-sequencing was carried out on the HiSeq 2500 platform (Illumina) at the Next-Generation Sequencing Core at the University of Texas MD Anderson Cancer Center Science Park. The Bioo Kit Option 2 protocol was utilized to prepare sequencing libraries, with 1.7–10 ng of DNA per sample.

DNA isolated via ChIP was then analyzed by qPCR using SYBR Green Master Mix on Bio-Rad CFX96 real-time PCR system. Primers were designed to hybridize at the transcriptional start sites that exhibited differential DNA enrichment between VEH- and EDC-MMSCs; the sequences of the primers are listed in Table S2. The results are presented as the percentage of the input DNA (% input) co-precipitated with anti-H3K4me3 or control antibody. The antibodies used for ChIP-seq are shown in Table S1.

### Epigenomics data analysis

ChIP-seq reads were trimmed for low-quality base pairs using TrimGalore. Data was mapped to the rat genome build UCSC rn6 using the bowtie2 software. Duplicate reads were removed, then ChIP-seq tracks were prepared using bedtools, normalized to reads per million reads mapped (rpm). ChIPseq tracks were visualized using the Integrative Genome Viewer software. Differential ChIP-seq regions were determined using the diffReps software using the Gtest, with significance achieved for a fold change exceeding 1.5 × , and an FDR-adjusted *q* value < 0.01. Differential regions were annotated for nearby genes using BEDTOOLS; specifically, we considered genes with a differential region within 3 kb from its TSS. Venn diagram analysis of genes associated with differential peaks was carried out using the Python language scientific library. ChromHMM was used with the H3K4me3 ChIP-seq data to generate an epigenomic states partition of the rat MMSC epigenome. Epigenomic states were annotated based on the emission matrix following the approaches used by the Encode and the NIH Epigenome Roadmap consortia. Overlap of differential regions with epigenomic status was determined using BEDTOOLS. Odds-ratio enrichments for individual epigenomic states were then computed based on the cumulative genome-wise size of each epigenomic state. Odds ratios were graphed using GraphPad Prism 8.02.

### Reduced representation bisulfite sequencing and targeted next-generation sequencing

Reduced Representation Bisulfite Sequencing (RRBS) libraries were constructed using the RRBS Methyl-Seq kit from Nugen (San Carlos, CA, USA). Briefly, 100 ng of high-molecular-weight DNA was digested with *MspI*, ligated to sequencing adaptors, treated with bisulfite, and amplified by PCR. The final libraries were quantitated with Qubit (ThermoFisher Scientific), and the average size was determined on a Fragment Analyzer (Agilent, CA). The libraries were diluted to 10 nM and further quantitated by qPCR on a CFX Connect Real-Time qPCR system (Bio-Rad, Hercules, CA, USA) for accurate pooling of barcoded libraries and maximization of the number of clusters in the flowcell. Quantitated libraries were prepared for sequencing on a NextSeq MO flowcell according to Illumina recommendations. The final prepared libraries were loaded on the flowcell at 2 pM, and sequencing was performed with single reads of 100 bp with a 12 bp index read.

For targeted next-generation sequencing (NGS), amplicon sequencing on the NGS platform was used to determine the DNA methylation status of the promoter regions of ERGs as previously described [36]. Genomic DNA samples from VEH-MMSCs and EDC-MMSCs in each experimental group were isolated using the Puregene core Kit (Qiagen) and purified using nucleic acid purification columns (Qiagen). Genomic DNAs were treated with sodium bisulfite as previously described [37], and PCR amplifications were performed using the primers listed in Table S2. The locations of CpG sites are shown in Table S3. The PCR products were sequenced on Ion Torrent PGM, and raw sequence data were imported into the software package CLC genomics workbench (CLC bio, Qiagen). Data were quality trimmed (0.01 or Q20), and primer and linker sequences were removed using the trimming algorithm implemented within CLC genomics. Subsequently, the trimmed data were mapped against the reference converted sequence, assuming no methylation. Variant calling using the quality variant algorithm was performed using a minimum threshold of 0.1%. A table of variants relative to the reference was generated, and known CpG sites were identified. The percentage of methylation was determined by comparing variant abundance values for each site.

### Western Blot analysis

Western blot analysis was performed as previously described [26]. The levels of targeted proteins were measured in three biological replicates. The antibodies used in this study are listed in Table S1.

### Knockdown of *Tasp1* in MMSCs from Eker rats

To determine methyltransferase specificity for changes in H3K4me3 in response to EDC, we conducted a transient knockdown of Taspase1 (*Tasp1*) expression. This was achieved using viral particles from three rat shRNA constructs (OriGene, Rockville, MD, USA). EDC-MMSCs were cultured in 6-well plates and incubated with lentiviral particles at a virus multiplicity of infection (MOI = 10) overnight. Subsequently, the medium was replaced, and the cells were allowed to grow for five days. Lysates and RNA were then prepared from the cells and used to measure the protein and RNA levels in MMSCs with *Tasp1* rat shRNA lentiviral particles or scrambled controls.

### Secretome from MMSCs and its effect on DMCs characteristics

Conditioned medium from MMSCs was prepared as previously described [38]. Differentiated Myometrial Cells (DMCs) were isolated from rat adult myometrium using our previous methods [39]. To determine the paracrine effect of reprogrammed MMSCs on DMCs, the DMCs were grown in the presence of conditioned medium collected from either EDC-MMSCs or VEH-MMSCs for two days, before the performance of the assays.

### MTT assay

The MTT cell proliferation assay was performed as previously described [33].

### Statistical analysis and bioinformatics analysis

Student’s *t* test (GraphPad Prism) was used to determine whether there were significant differences between VEH and EDC treatment groups for gene expression (measured by real-time RT-qPCR), fluorescent signals (measured by immunofluorescence microscope), protein levels (measured by Western blot), luciferase activity (measured by luciferase activity kit), and histone modification (measured by ChIP-qPCR). *p* ≤ 0.05 was considered significant.

Fisher’s exact *t* test was done to examine the correlation between EDC-regulated genes and the H3K4me3 enrichment/DNA methylation status of reprogrammed genes. In addition, the Wilcoxon test was used to examine the statistical significance in DNA methylation levels between EDC- and VEH-MMSCs.

Top biological functions and canonical pathways associated with the differentially expressed mRNA data set were identified with Ingenuity Pathway Analysis (IPA) (Qiagen). A *z*‐score was calculated to infer the activation states of implicated biological processes. Over-representation Analysis (ORA) was used to determine over-represented biological processes in our experimentally derived gene list. The *p* value is calculated by hypergeometric distribution.

## Results

### Identification of a distinct transcriptional pattern in EDC- vs. VEH-exposed MMSCs of Eker rats

**Fig. 1:**
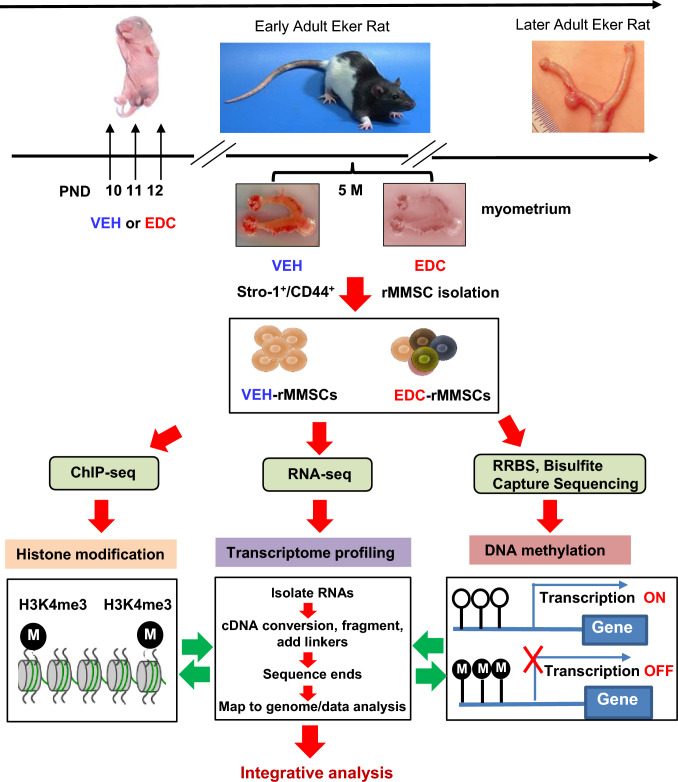
Experimental paradigm. Eker rat pups were exposed to VEH and EDC diethylstilbestrol at postnatal days 10-12. The pups were sacrificed at 5 months of age representing the early adult stage. Myometrial tissues were isolated from the animals and subjected to MMSC isolation using Stro-1/CD44 surface markers. Myometrium from five animals was pooled for each treatment. Multi-omics analyses, including RNA-sequencing, ChIP-sequencing, and RRBS, were performed to determine the global alterations of the transcriptome, histone modification, and DNA methylation, respectively. In addition, targeted bisulfite NGS was performed to examine the DNA methylation within CpG islands of genes

**Fig. 2:**
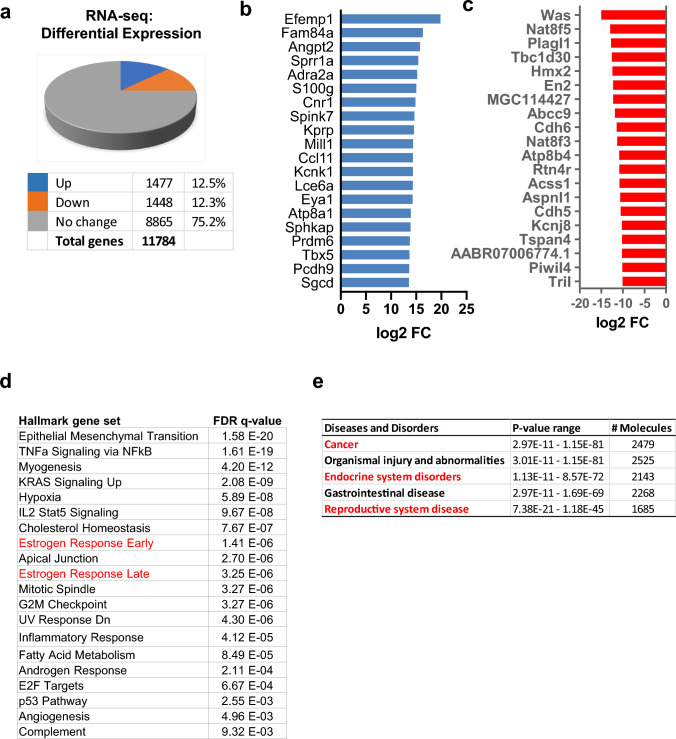
Developmental EDC-induced reprogramming of rat MMSCs genes. **a** Five animals from each of the EDC diethylstilbestrol and VEH groups were pooled and subjected to MMSC isolation using a FACS strategy. Pie chart showing the percentage of genes that exhibited changes in RNA expression between EDC-MMSCs and VEH-MMSCs as measured by RNA-seq; the cutoff value is twofold with an FDR < 0.05. **b** List of the top 20 up-DEGs in EDC- vs. VEH-MMSCs. **c** List of the top 20 down-DEGs in EDC- vs. VEH-MMSCs. **d** Over-representation analysis showed that multiple UF-related pathways, including the estrogen response pathways (red color) were affected by early-life EDC exposure. **e** Relevant diseases affected by developmental EDC diethylstilbestrol exposure were analyzed by Ingenuity Pathway Analysis (IPA) and highlighted in red color

To identify differences in gene expression that drive the pathogenesis of UFs in the context of early-life EDC diethylstilbestrol (DES) exposure, we used the Eker rat model [32]. Neonatal rats were exposed to EDC or VEH at postnatal days 10–12. At five months of age, we collected myometrial tissues from the animals and isolated rMMSCs, the cell of origin of UFs, using Stro-1 and CD44 surface markers (see the experimental design in Fig. 1). By RNA-sequencing (RNA-seq), we found 2922 differentially expressed genes (DEGs) between EDC-MMSCs and VEH-MMSCs among 11,784 genes detected (Fig. 2a). Among the DEGs, 1477 were upregulated, while 1448 genes were downregulated (Fig. 2a). The top 20 up- and downregulated genes in EDC-MMSCs compared to VEH-MMSCs are listed in Fig. 2b and c, respectively.

ORA using Hallmark Gene Sets showed that genes whose expression was altered by EDC were enriched for multiple cellular pathways, including but not limited to estrogen response, metabolism, inflammatory response, androgen response, and hypoxia (Fig. 2d). These cellular pathways have been shown to play important roles in the pathogenesis of many diseases, including reproductive diseases such as UFs. Ingenuity Pathway Analysis (IPA) of DEGs identified an association of EDC-regulated genes with cancer, endocrine disorders, and reproductive disease (Fig. 2e).

### Developmental EDC exposure reprograms estrogen-responsive genes in MMSCs

**Fig. 3:**
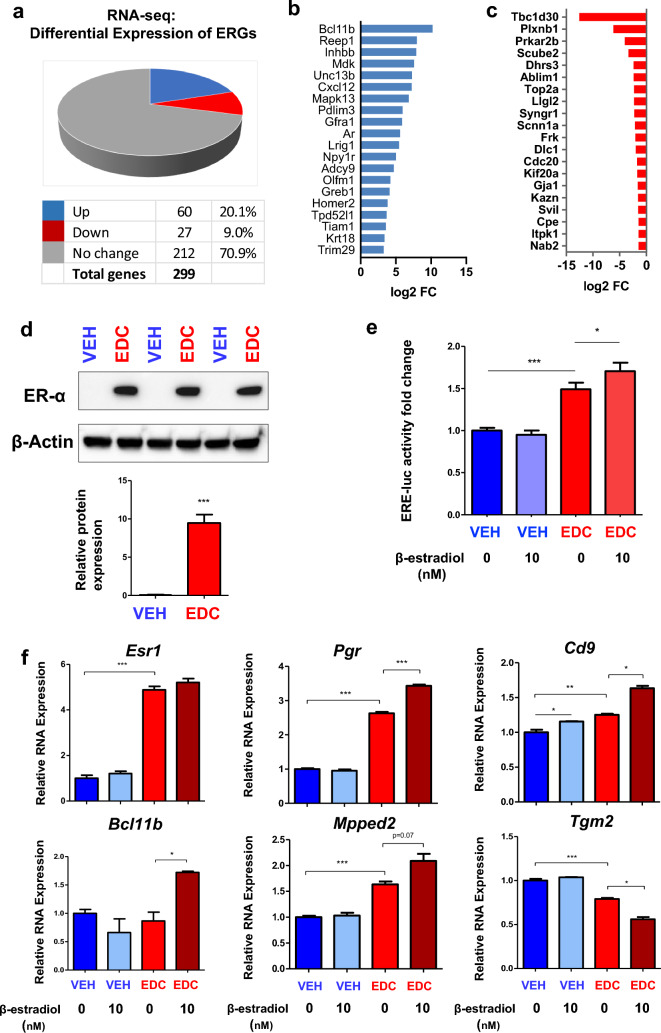
Transcriptional profiling reveals reprogramming of estrogen-responsive gene. **a** Pie chart showing the percentage of ERGs that exhibited changes in RNA expression in EDC-MMSCs compared to VEH-MMSCs, as measured by RNA-seq. **b** List of the top 20 up-ERGs, that showed differential expression as measured by RNA-seq in EDC- vs. VEH-MMSCs. **c** List of the top 20 down-ERGs, that showed differential expression as measured by RNA-seq in EDC- vs. VEH-MMSCs. **d** ER-a is upregulated in EDC-MMSCs compared to VEH-MMSCs. Lysates were prepared from EDC-MMSCs and VEH-MMSCs and subjected to Western blot analysis using antibody against ER-a. **e** Luciferase activities in the presence or absence of estrogen (10 nM) were compared between EDC-MMSCs and VEH-MMSCs. **f** Alteration of ERG expression in the presence or absence of estrogen in DES-MMSCs and VEH-MMSCs. Student’s *t*-test, *p < 0.05; **p < 0.01; ***p < 0.001

Endocrine disruptors, including DES, are substances that interfere with the actions of natural hormones, such as estradiol, in the body responsible for development. Since the natural hormone of estradiol acts via several mechanisms, we focused on the effect of EDC exposure on estrogen responsiveness. Therefore, 299 ERGs from the Hallmark (MSigDB) early estrogen response and late estrogen response pathways were examined in EDC-MMSCs and VEH-MMSCs. We identified 87 ERGs whose gene expression was dysregulated in EDC-MMSCs compared to VEH-MMSCs. Among them, 60 ERGs were upregulated, and 27 ERGs were downregulated in EDC-MMSCs compared to VEH-MMSCs (Fig. 3a). The top 20 upregulated and downregulated ERGs in EDC-MMSCs compared to VEH-MMSCs are shown in Fig. 3b and c respectively. The overlap of EDC-regulated genes in MMSCs with estrogen early and late response genes is shown in the Venn diagrams (Fig. S1).

Since one of the main estrogenic activities is through estrogen receptor (ER), we determined the protein levels of ER and demonstrated that EDC exposure dramatically increased the ER protein levels in EDC-MMSCs compared to VEH-MMSCs (Fig. 3d, *p* < 0.001), suggesting that ER-mediated signaling might be activated due to early-life exposure to EDC. To test this hypothesis, we infected the EDC-MMSCs and VEH-MMSCs with *ad-ERE-luc* and measured the luciferase activity of EDC-MMSCs and VEH-MMSCs. As shown in Fig. 3e, the luciferase activity driven by estrogen-responsive element (ERE) is significantly higher in EDC-MMSCs than VEH-MMSCs. Notably, adding 10 nM estradiol further increased the luciferase activity in EDC-MMSCs but not in VEH-MMSCs. These studies confirmed our hypothesis that EDC exposure enhances estradiol action via increased ER levels. Next, we determined if estradiol treatment increased the expression levels of ERGs in EDC-MMSCs. As expected, estradiol treatment at 10 nM significantly increased the expression of 4 ERGs, including *Pgr*, *Cd9*, *Bcl11b*, and *Mpped2.* In contrast, the expression of *Pgr*, *Bcl11b*, *and Mpped2* showed no changes, while *Cd9* expression was increased, in VEH-MMSCs after estradiol treatment. Interestingly, we also observed that while the expression of *Pgr*, *Cd9*, and *Mpped2* was higher in EDC-MMSCs (experimental group) than VEH-MMSCs when grown in an estradiol-free medium, the fold changes in the expression of these ERGs were more significant in EDC-MMSCs when grown in an estradiol-containing medium (Fig. 3f). Notably, the expression of *Esr1* was not changed in response to estradiol treatment in both EDC-MMSC and VEH-MMSCs. In contrast to the increased expression of ERGs (*Pgr*, *Cd9*, *Bcl11b*, and *Mpped2*) in EDC-MMSCs following estradiol treatment, estradiol downregulated the expression of *Tgm2* encoding transglutaminase 2, which catalyzes the cross-linking of proteins, in EDC-MMSCs. These studies suggested that developmental EDC exposure reprogrammed the estrogen signaling pathway leading to hyper-estrogenic responsiveness in MMSCs.

### EDC exposure activates MLL1 epigenetic pathways and disrupts epigenome via H3K4me3 in MMSCs

**Fig. 4:**
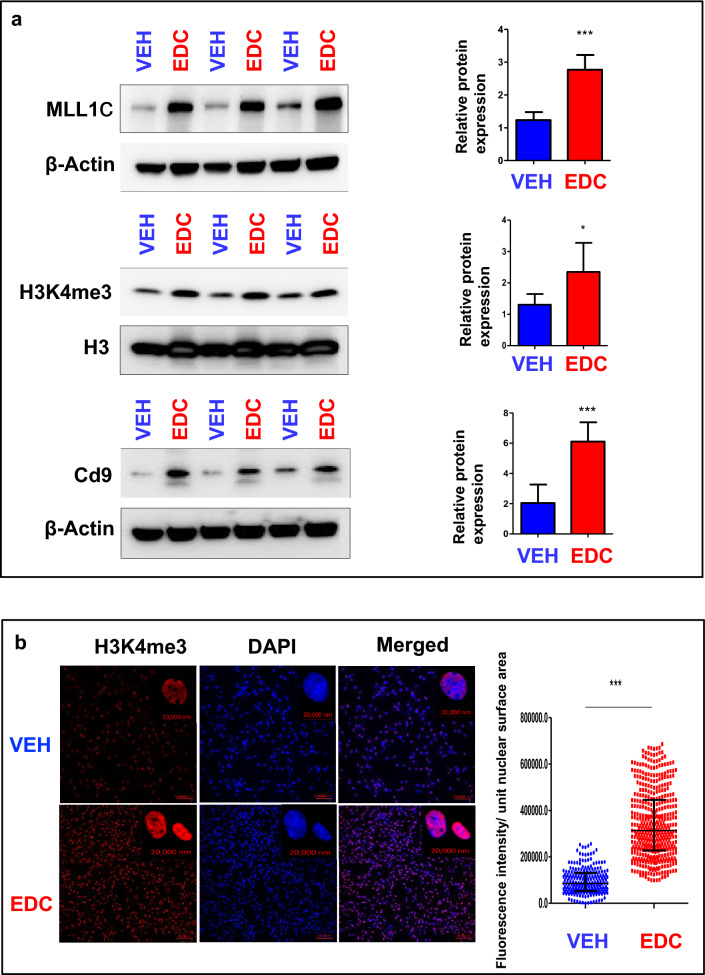
Developmental exposure to EDC activates MLL1. **a** Western blotting with anti-MLL1, -H3K4me3, and -Cd9 antibodies was performed to determine the levels of MLL1C (the activated form of MLL1), H3K4me3 and CD9. Total H3 and β-actin were used as loading controls. Biological replicates (*n* = 3) were used for quantification. **b** Confocal imaging showing the staining of H3K4me3 in EDC- vs. VEH-MMSCs and quantitative analysis. Three separate coverslips of cells were used for quantification. Student’s *t*-test, *p < 0.05; **p < 0.01; ***p < 0.001

After discovering that exposure to EDC during the neonatal stage significantly modified the gene expression pattern of ERGs, we next sought to identify the underlying mechanism responsible for these EDC-induced changes in gene expression. In our investigation, we turned our attention to Mixed-lineage Leukemia1 (MLL1), an extensively studied mammalian counterpart of the yeast protein Set1, which is a component of the COMPASS complex (complex of proteins associated with Set1) (see Figure S2a, left panel). MLL1 is an initial 500-kDa protein cleaved by Taspase1 (Tasp1) to produce a mature MLL1 N320/C180 heterodimer [34]. Specifically, we focused on Mixed-lineage Leukemia1 (MLL1 C180), which has a conserved SET domain that methylates histone H3 at K4, resulting in the activation-associated H3K4me3 modification (Fig. S2, right panel). To investigate the involvement of MLL1-mediated epigenetic regulation, we examined the expression levels of H3K4me3 (a histone modification) and the C-terminal fragment of MLL1 in EDC-MMSCs compared to VEH-MMSCs. We observed a significant increase in the cleavage and formation of activated MLL1 in EDC-treated MMSCs, which correlated with elevated levels of H3K4me3 (Fig. 4a). Notably, we also observed an upregulation of CD9 protein, which is encoded by the Cd9 gene that undergoes reprogramming through H3K4me3 modification (Fig. 4a). Confocal images further demonstrated by immunostaining a substantial increase in H3K4me3 levels in EDC-MMSCs compared to VEH-MMSCs (Fig. 4b, left and right panel, *p* < 0.001).

**Fig. 5:**
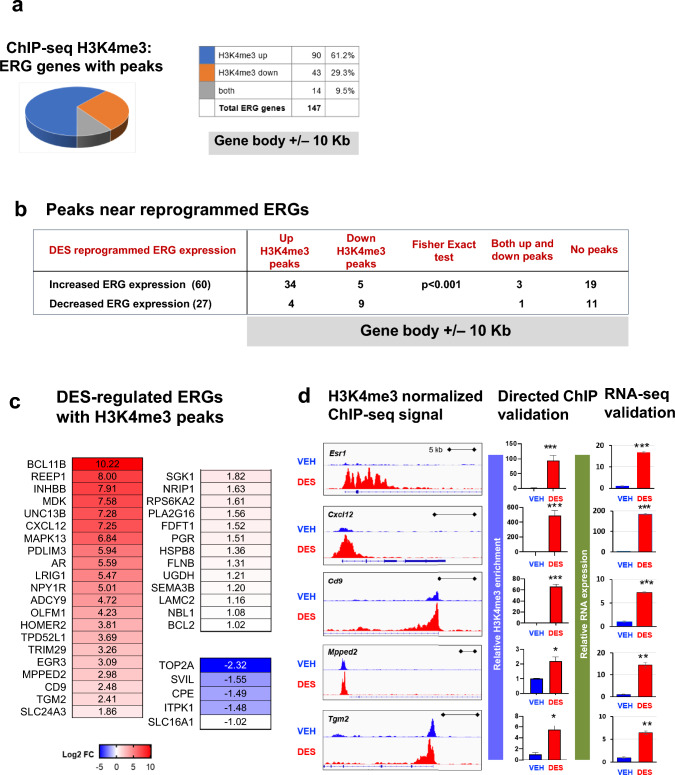
EDC exposure disrupted the epigenome in MMSCs. **a** Pie chart showing the number of ERGs with peaks. **b** Integration of H3K4me3 with RNA expression of ERGs by EDC exposure. **c** EDC-regulated ERGs with H3K4me3 peaks. **d** Histograms from Integrative Genomics Viewer showing H3K4me3 occupancy at *Esr-1, Cxcl12*, *Cd9*, *Mpped2, and Tgm2* (left panel). For each gene, the upper and the lower browser images display an expanded view of a selected region of the H3K4me3 peak distributions in VEH-MMSCs (blue track) and EDC-MMSCs (red track). Middle panel: directed ChIP-sequencing validation of H3K4me3 target genes by ChIP-qPCR; right panel; RNA-sequencing validation by q-PCR. Student’s *t*-test, *p < 0.05; **p < 0.01; ***p < 0.001

To determine whether MLL1 plays a role in regulating the expression of ERGs after EDC exposure, whole-genome ChIP-sequencing (ChIP-seq) for H3K4me3 was performed. The results, depicted in Fig. 5a, revealed that in EDC-MMSCs compared to VEHMMSCs, H3K4me3 was enriched at 90 peaks (61.2%) associated with ERGs, while it was reduced at 43 peaks (29.3%). We next integrated the gene expression data from RNA-seq with H3K4me3 enrichment in EDC- and VEH-MMSCs. As shown in Fig. 5b, we observed a significant correlation between ERG expression and the H3K4me3 status. Specifically, genes with upregulated H3K4me3 displayed increased ERG expression compared to genes with downregulated H3K4me3. Furthermore, we also examined genes where H3K4me3 was upregulated or had both upregulated and downregulated regions within the gene body, along with a 10-kb window surrounding it (*p = 0.0002, Fisher’s exact test).

The EDC-regulated ERGs with H3K4me3 peaks were shown in Fig. 5c. Figure 5c lists the reprogrammed ERGs with increased or decreased H3K4me3 enrichment. Considering that estrogen receptor α (*Esr1*) serves as the upstream master regulator of ERG expression, we first determined whether developmental exposure to EDC reprograms the *Esr1* gene via H3K4me3. As shown in Fig. 5d, a marked increase in H3K4me3 enrichment at the *Esr1* gene was seen in EDC-MMSCs compared to VEH-MMSCs. Similar patterns of H3K4me3 enrichment were also found in other ERGs, including *Cxcl12, Cd9, Mpped2, and Tgm2* (Fig. 5d). The enrichment of H3K4me3 at the promoter regions of these genes was validated by ChIP-qPCR (Fig. 5c, middle panel). In addition, RNA expression of these five ERGs (*Esr1*, *Cxcl12, Cd9, Mpped2* and *Tgm2)* was validated by qPCR (Fig. 5d, right panel), which is consistent with RNA-seq data.

**Fig. 6:**
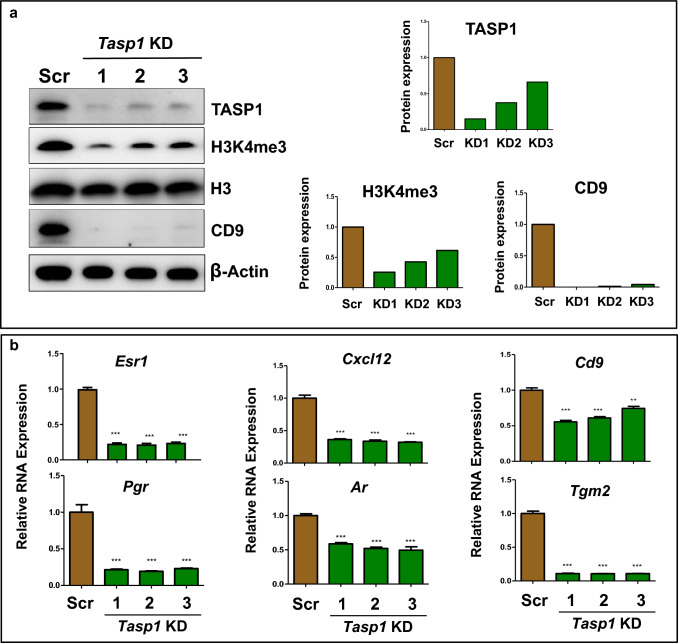
Knockdown of *Tasp1* reversed the reprogrammed ERGs induced by EDC exposure. **a** Western blot analysis was conducted to determine the role of TASP1 in MLL1-mediated epigenetic pathways after knock-down of *Tasp1* expression using shRNA lentiviral (pLKD-TASP1) plasmids in EDC-MMSCs. The levels of expression of TASP1, H3K4me3, and CD9 proteins were measured by Western blot analysis using antibodies against TASP1, H3K4me3 and CD9, respectively (left panel). Biological replicates (*n* = 3) were used for quantification. Quantitative analysis was performed using Image J (right panel). **b** the upregulation of the expression of 6 reprogrammed ERGs (*Esr-1, Pgr, Cxcl12, Ar, Cd9, and Tgm2*) induced by EDC exposure was reversed by *Tasp1* knock-down in EDC-MMSCs. RNA expression of ERGs was measured by qPCR in EDC-MMSCs infected with either scrambled lentivirus or one of three different individual *Tasp1* knock-down lentiviruses. *p < 0.05; **p < 0.01; ***p < 0.001; Student’s *t*-test

Based on these findings, we hypothesized that blocking the cleavage of MLL1, mediated by Tasp1, would reverse the increased expression of reprogrammed ERGs by EDC exposure. To address this hypothesis, we knocked down *Tasp1* in EDC-MMSCs using a lentiviral shRNA approach. As seen in Fig. 6a, the Western blot analysis demonstrated a significant decrease in *Tasp1* expression upon transfection with three different *Tasp1* shRNAs. As expected, global levels of H3K4me3 were decreased upon *Tasp1* knockdown. Notably, protein expression of the H3K4me3-reprogrammed gene *Cd9* was remarkably reduced (> 95%). We next measured the RNA expression of H3K4me3-reprogrammed ERGs after *Tasp1* knockdown. As shown in Fig. 6b, the knockdown of *Tasp1* led to a substantial reduction in the expression of EDC-reprogrammed ERGs, including *Cd9*, *Pgr*, *Ar*, *Cxcl12*, and *Tgm2 as well as Esr1.* These results suggest that activated MLL1 plays a crucial role in reprogramming ERGs, leading to increased expression of ERGs in EDC-exposed MMSCs.

### EDC exposure reprograms the methylome and alters ERG expression.

**Fig. 7:**
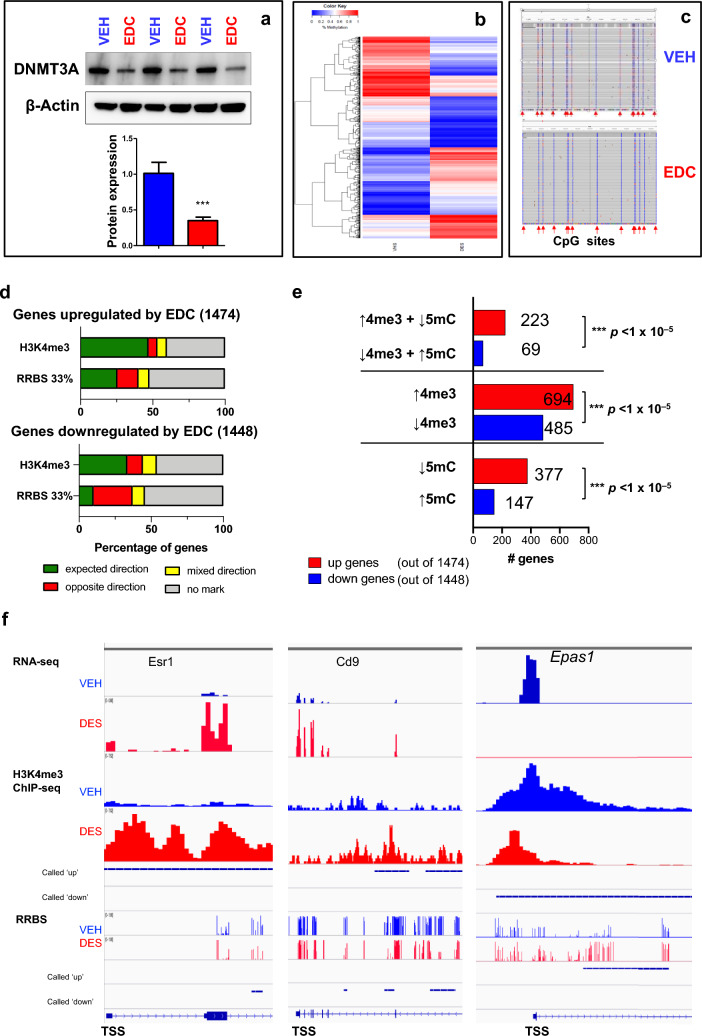
Developmental EDC exposure alters the methylome and reprograms ERGs via DNA promoter methylation. **a** Western blot analysis was performed to measure the protein levels of DNMT3A in VEH-MMSCs and EDC-MMSCs. **b** Heatmap of genes with DNA methylation in VEH- and EDC-MMSCs. **c** The methylation tracks of *Esr1* determined by targeted bisulfite NGS, which covered 14 CpG sites located in its CpG island. **d** Percentage of EDC-regulated genes with status of H3K4me3 and DNA methylation. **e** Number of genes with status of H3K4me3 and DNA methylation*.* **f** Integrated genome viewer (IGV) plots and integration of multi-omic analysis. Peaks with H3K4me3 enrichment, DNA methylation, and RNA expression in EDC-MMSCs are indicated in red, while peaks with H3K4me3 enrichment, DNA methylation, and RNA expression in VEH-MMSCs are indicated in blue. Student’s *t*-test, ***p < 0.001

Since DNA methylation is another key mechanism through which gene expression is regulated, we next determined whether developmental exposure to EDC alters ERG expression through altered DNA methylation status. Western blot analysis demonstrated that the protein levels of DNMT3A were significantly decreased in EDC-MMSCs as compared to VEH-MMSCs (Fig. 7a). These data indicated that EDC exposure may alter the methylome in MMSCs. Therefore, we conducted a global Reduced Representation Bisulfite Sequencing (RRBS) analysis in which we compared the DNA methylation in EDC-MMSCs and VEH-MMSCs genome-wide. A total of 1,654,267 (minimum 5X in both samples) CpG sites were analyzed. Figure 7b exhibited a differential methylation pattern between EDC-MMSCs and VEH-MMSCs, suggesting that EDC may regulate gene expression in MMSCs via DNA methylation.

Regarding the genes upregulated by EDC exposure, when applying a methylation threshold of 33%, approximately 25.58% displayed the expected mark, 14.59% exhibited the opposite mark, 7.53% showed mixed directions, and 52.15% did not exhibit any methylation mark (Figure S3). On the other hand, for the genes downregulated by EDC exposure, approximately 10.15% displayed the expected mark, 27% showed the opposite mark, 8.49% exhibited mixed directions, and 54.35% did not exhibit any methylation mark (Figure S3). The correlation between EDC-regulated gene expression and DNA methylation with 10% and 50% thresholds is also shown in Figure S3.

Since CpG island methylation status is linked to gene expression, we next used the targeted bisulfite NGS approach to measure the DNA methylation levels at the CpG islands of *Esr1*, the master of estrogen signaling. As shown in Fig. 7c, all the 14 CpG sites in the promoter region of *Esr1* exhibited hypo-methylation in EDC-MMSCs compared to VEH-MMSCs, suggesting reduced DNA methylation at the CpG island of *Esr1* may contribute to increased *Esr1* expression in EDC-MMSCs (Figure S3a).

We then determined whether other ERGs that exhibited enrichment of H3K4me3 were differentially methylated. Six ERGs, including *Ar*, *Cxcl12*, *Cd9*, *Bcl11b*, *Mpped2*, and *Tgm2*, revealed hypo-methylation within the CpG islands in their promoter regions. Of the 17 CpG sites examined in *Ar*, all but one (Chr X: 68534849, rn5) showed decreased methylation levels in EDC-MMSCs compared to VEH-MMSCs (*p* < 0.05). Similar decreases in methylation levels were found in other ERGs, including *Cxcl12*, *Cd9*, *Bcl11b*, *Mpped2*, and *Tgm2*. Moreover, we measured the DNA methylation levels of the *Pgr* promoter regions and observed hypo-methylation of 7/19 (36.8%) CpG sites in the promoter region. The methylation levels of *Cxcl12* and *Pgr* were lower in both EDC-MMSCs and VEH-MMSCs. Wilcoxon test analysis showed that the DNA methylation levels of *Esr1* and those of 7 ERGs covering 102 CpG sites exhibited a significant difference between EDC- and VEH-MMSCs (*p* < 0.05) (Fig. S3).

Integration analysis of RRBS, ChIP-seq, and RNA-seq demonstrated that among 1474 EDC-upregulated genes with expected direction, 47.1% of genes were correlated with H3K4me3 enrichment. We also observed that 25.6% of genes with DNA hypo-methylation were associated with EDC-induced upregulation of RNA expression using a 33% methylation threshold. Among 1448 EDC-down regulated genes, 33.5% of genes associated with H3K4me3 reduction. We also observed that 10.2% of genes with DNA hyper-methylation correlated with EDC-down-regulated genes (Figs. 7d, S3).

In terms of the analysis for opposite direction effects, we observed that only 6.11% of EDC-upregulated genes correlated with H3K4me3 reduction. Furthermore, we found that 14.6% of EDC-upregulated genes associated with increased methylation status using 33% methylation thresholds. For EDC-downregulated genes, only 10.8% of EDC-regulated genes correlated with H3K4me3 enrichment. However, 27% of genes correlated with reduction of DNA methylation using 33% methylation thresholds. Notably, we observed that 40.2% of EDC-upregulated genes and 46.1% of EDC-downregulated genes showed no H3K4me3 mark (Figs. 7d, S3), suggesting that other mechanisms are involved in regulating gene expression in MMSCs by EDC exposure. In addition, 6.6% of EDC-upregulated genes showed both H3K4me3 enrichment and reduction. On the other hand, 9.5% of EDC-down-regulated genes showed both H3K4me3 enrichment and reduction (Fig. S4).

Considering expected direction analysis, the status of H3K4me3 (enrichment or reduction) plays a dominant role in regulating gene expression in MMSCs genome-widely in response to EDC exposure. We also noticed that both DNA methylation and H3K4me3 co-regulate some of the same targets in response to EDC exposure. As shown in Fig. S4, 23.1% of EDC-upregulated genes exhibited overlap between H3K4me3 and RRBS using 33% methylation thresholds. On the other hand, 11.5% of EDC-down-regulated genes showed an overlap between H3K4me3 and RRBS. We also compared the number of genes with the integration of DNA methylation and H3K4me3 status. We found a significant difference between the number of genes exhibiting H3K4me3 enrichment and hypo-methylation versus those showing H3K4me3 reduction and hyper-methylation (Fig. 7e, *p* < 0.001). Similar findings with significant differences were seen in a comparison of regulated gene numbers between the H3K4me3 enrichment and reduction group, as well as between the hypo-methylation versus hyper-methylation group (Fig. 7e).

The status of H3K4me3 enrichment, DNA methylation, and RNA expression of *Esr1*, *Cd9*, and *Epas1* genes in EDC-MMSCs and VEH-MMSCs is shown in Fig. 7f. For the *Esr1* gene, the H3K4me3 enrichment correlated with increased RNA expression in EDC-MMSCs compared to VEH-MMSCs with reduction in DNA methylation. A similar finding was observed for the *Cd9* gene. For *Epas1*, the reduction of H3K4me3 correlated with increased methylation around TSS region, concomitantly with decreased expression of *Epas1* in EDC-MMSCs (Fig. 7f). These data suggested that the expression of *Esr1*, *Cd9*, and *Epas1* genes was altered by early-life exposure to EDC via DNA methylation and chromatin regulation mechanisms (H3K4me3 enrichment or reduction).

**Fig. 8:**
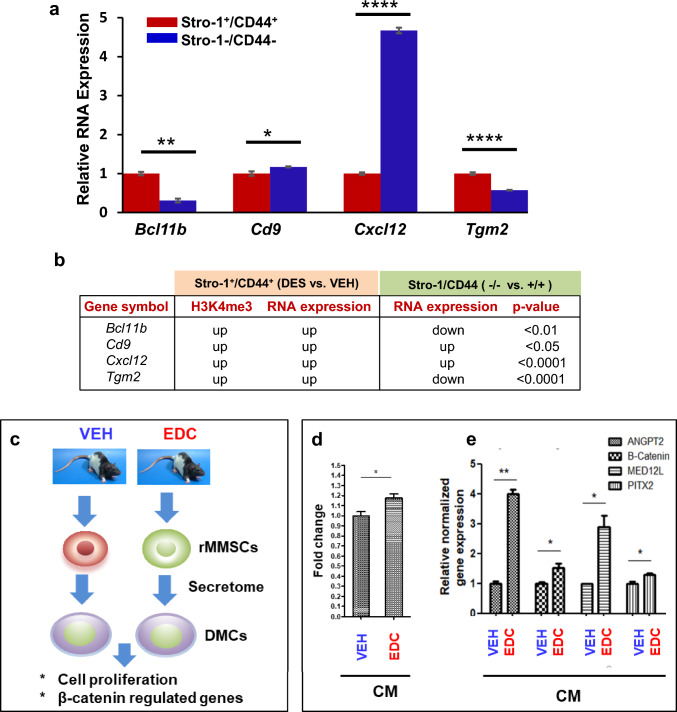
Specific reprogramming of ERGs in DES-MMSCs, which impacts DMC. **a** Bar plots showing the differential expression of ERGs, including *Bcl11b*, *Cd9*, *Cxcl12*, and *Tgm2*, between DES-MMSCs and DES-DMCs. **b** The correlation between H3K4me3 status and compared expressions of reprogrammed ERGs between MMSCs and DMCs using VEH-MMSC as a reference. The p-value shows the significant difference in gene expression between Stro-1/CD44 double-positive and double-negative cells. The genes with light blue background indicate that two comparisons of the gene expression (Stro-1/CD44 double-positive vs. double-negative cells, or Stro-1^+^/CD44^+^ DES vs. VEH) going in the opposite direction. The genes with white background indicate that two comparisons go in the same direction. **c** Diagram of experimental design. Serum-free conditioned medium (CM) was prepared from EDC-MMSCs and VEH-MMSCs. DMCs from rat adult myometrium were grown in the CM from EDC-MMSCs and VEH-MMSCs for 2 days. **d** MTT assays were performed to measure DMC proliferation in the presence of CM from either EDC-MMSCs or VEH-MMSCs. **e** qPCR was performed to determine the effect of CM from EDC-MMSCs and VEH-MMSCs on the expression of β-catenin and β-catenin-regulated genes (*Angpt2*, *Med12l*, and *Pitx2*) in DMCs. *p < 0.05; **p < 0.01, ****p < 0.0001

To determine if ERG reprogramming via early-life exposure to DES is MMSC-specific, we compared the expression of ERGs between the MMSCs and differential myometrial cells (DMCs, Stro-1^−^/CD44^−^) from the myometria of Eker rats developmentally exposed to DES. As shown in Fig, 8a, out of four ERGs (*Bcl11b, Cd8, Cxcl12, and Tgm2*) with enriched H3K4me3, all were significantly altered. Notably, two genes (*Bcl11b and Tgm2*) showed downregulated expression in DES-exposed DMCs compared with DES-exposed MMSCs, while the other ERGs (*Cd9* and *Cxcl12*) exhibited upregulation in DMCs compared to MMSCs. Given the fact that these four genes are upregulated in DES-MMSCs compared to VEH-MMSC, one may consider that some of the reprogrammed ERGs are MMSC-specific (Fig. 8b).

### Reprogrammed MMSCs alters the surrounding differentiated myometrial cells

To determine whether reprogrammed MMSCs affected the characteristics of surrounding differentiated myometrial cells (DMCs), we isolated adult rat DMCs from normal myometrium and exposed them to conditioned medium (CM) from VEH-MMSCs or EDC-MMSCs (Fig. 8c). First, we determined whether the EDC-exposed MMSCs altered the cellular behavior of DMCs. CM from EDC-exposed MMSCs enhanced the proliferation of DMCs compared to CM from VEH MMSCs by 20% (Fig. 8d). Importantly, EDC-exposed MMSCs activated β-catenin signaling in DMCs. The expression of β-catenin and β-catenin-regulated genes (*Angpt2*, *Med12l*, and *Pitx2)* was upregulated in DMCs in the presence of CM from EDC-MMSCs as compared to CM from VEH-MMSCs (Fig. 8e).

## Discussion

Accumulating evidence demonstrates that environmental exposures to EDCs can reprogram the epigenome of developing tissues in such a way as to increase susceptibility to disease later in life [4, 9, 40, 41]. For example, increased carcinogenesis and epigenetic reprogramming at DNA methylation and histone modification levels, in response to early-life exposure to EDC bisphenol A (BPA) have also been demonstrated for prostate tumors [4, 34, 42, 43]. Notably, a previous study demonstrated that BPA could promote human prostate stem-progenitor cell self-renewal and increased in vivo carcinogenesis in the human prostate epithelium [44]. These data are consistent with our recent finding that exposure to EDC in early life promotes the expansion of MMSCs. This phenomenon is associated with an increased risk of UF pathogenesis [7] and emphasizes the critical role of tissue stem cells in the developmental origin of adult diseases. However, the mechanism by which developmental exposure to EDCs increases the risk of adult disease at the level of tissue stem cells in EDC-targeted tissues is unknown [6, 41].

Here, we used Eker rats that carry a germ-line mutation in the tuberous sclerosis complex 2 (*Tsc2*) tumor suppressor gene and are susceptible to the development of UFs. By isolating myometrial Stro1^+^/CD44^+^ stem cells (MMSCs), the type of cells from which potential UFs originate, we could examine the effect of early-life exposure to EDC through multi-omics analyses. Our proof-of-concept using combined ChIP-seq, DNA methylation, and RNA-seq analyses in this model demonstrates that both DNA methylation and histone modification at H3K4me3 in MMSCs, are highly associated with the increased expression of ERGs that have been linked to increased risk of UFs.

To investigate how exposure to EDC during a critical period of uterine development increases the likelihood of developing UFs, we conducted multi-omics studies using MMSCs exposed to EDC during a critical period of uterine development. Our findings demonstrate that developmental exposure to EDC during uterine development alters the normal response of targets in MMSCs. We observed specific changes in the programming of enriched ERGs in MMSCs following exposure to EDC, before the development of tumors. Our experimental design provides a unique opportunity to characterize alterations in the epigenome in MMSCs from 5-month-old animals (representing the early adulthood phase) before UF development, which typically occurs in late adult/old age in response to developmental EDC exposure (Fig. 1). This allows us to address the issue related to the window of transformation from normal to tumor status at the molecular level in MMSCs.

Changes in the gene expression of both known uterine developmental regulators (*Esr1* and *Pgr*), as well as newly characterized regulators, such as *Cd9* and *Cxcl12*, were identified. Over-representation analysis revealed the signaling pathways that were altered in EDC-exposed MMSCs, including estrogen response, androgen response, Inflammation, apoptosis, hypoxia, and metabolism, among others. Crucially, the expression of numerous estrogen-responsive genes (ERGs) associated with both estrogen activity and disease progression significantly rose due to exposure to EDC during early life. Similarly, like stem cells found in other hormone-dependent tissues, such as the breast and prostate [44], MMSCs naturally had low levels of estrogen receptor (ER) expression [45]. The low levels of ER expression could be considered as a protective mechanism against UF development. However, the early-life exposure to EDC disrupted this protective mechanism by increasing ER levels in MMSCs and promoting estrogenic effects. In contrast, estradiol did not enhance the levels of ER in MMSC from adult Eker rats without DES exposure. This DES-induced disruption resulted in the reprogramming of ER and ERGs, thereby enhancing estrogenic action in MMSCs. The primed MMSCs induced by early-life exposure to EDCs are vulnerable to insult exposure at a later stage. Estradiol treatment showed an exaggerated effect on the expression of ERG in DES-exposed MMSCs. This novel finding identified a clear link between EDC exposure and the risk of UFs at the stem cell levels via estrogen signaling. By IPA analysis in the disease and biological function category, EDC exposure was indeed linked to endocrine system disorders, reproductive system disease, and cancer. These studies strongly demonstrate that developmental exposure to EDC converts normal MMSCs to “hyper-estrogenic” MMSCs and provide evidence of the mechanistic link between altered epigenetic regulation by early-life EDC exposure and the latent onset of UF pathogenesis.

Furthermore, our research not only highlighted the significance of H3K4me3 in controlling the expression of genes associated with an elevated risk of UFs but also revealed that early-life exposure to EDC resulted in heightened levels of MLL1C (the active form of MLL-1) and H3K4me3 in MMSCs. TASP1, a protein responsible for regulating MLL-1 activity and the production of MLL1C, was found to be involved in this process. Importantly, when Tasp1 was knocked down, it reversed the EDC-induced increase in the expression of reprogrammed genes, indicating its crucial role in mediating the effects of EDC exposure. To our knowledge, this is the first demonstration that EDC exposure targets a histone methyltransferase, an enzyme that leads to epigenomic reprogramming in MMSCs. In a previous investigation focusing on prostate development, it was discovered that exposure to EDCs resulted in the activation of MLL1, leading to an increase in the presence of H3K4me3 at genes associated with prostate cancer. These changes persisted into adulthood. However, it is unclear how EDCs target the cells of origin in prostate carcinogenesis. In this study, we addressed this critical question related to the initiation of UFs. By isolating MMSCs from exposed and unexposed myometrium, we were able to identify specific epigenetic changes that occur in response to EDC exposure in MMSCs, the cells from which UFs originate. Here, we used DES as a proof of concept for EDC exposure. In a prior study, it was documented that exposing individuals to genistein, an EDC present in soybeans, during their developmental stages facilitated the growth of UFs, leading to an increase in both tumor incidence and multiplicity [9]. In this regard, our current research will help to understand better the molecular mechanism by which developmental EDC exposures increase UF development and provide a testable hypothesis for the prevention of UF development and progression. Moreover, these novel findings in this study will be helpful in providing insights into the occurrence of other hormonally dependent diseases, such as breast cancer and prostate cancer, whose “envirotypes” have not been fully characterized.

It is widely recognized that multi-omics analyses can provide a comprehensive understanding of the epigenomic network that regulates the transcriptomic landscape and the biological phenotype [46, 47]. In this study, in addition to histone modification and transcription analyses, we performed global DNA methylation analysis and targeted bisulfite sequencing to compare the methylation status of EDC-MMSCs with that of VEH-MMSCs. Developmental EDC exposure altered the methylome pattern and activated estrogen signaling, identified by reprogramming of ERGs via a DNA hypo-methylation mechanism. In addition, by combining these two genome-wide analyses into a set of ‘omics’, we identified the genotype that is linked to a specific envirotype. EDC could disrupt the epigenome of MMSCs via both histone modification and DNA methylation. Overlap of H3K4me3 and RRBS analyses demonstrated that these epigenetic mechanisms could modulate the same targets in MMSCs in response to EDC exposure. Furthermore, this is the first report indicating that the convergence of histone modification and DNA methylation actions on the same target genes, such as ERGs, makes MMSCs more vulnerable to environmental insults that promote the onset of UF in adulthood.

Comparing the epigenetic pattern between normal myometrial (MyoN) and myometrium from the uterus with UFs (MyoF) will help to determine the epigenetic risk signatures. A few studies [48–51] determined the DNA methylation pattern between human UFs and myometrium. Notably, hierarchical clustering based on DNA methylation can separate the myometrium and UFs. However, the DNA methylation pattern between MyoN and MyoF is similar [48]. Also, the enriched hypo-methylation of ERGs in our rat cell model cannot be observed in the comparison between MyoN vs. MyoF in humans. Although the comparison was done in different species, employing MMSCs could overcome the challenge of using heterogeneous myometrial tissues. Moreover, we compared the expression pattern of ERGs in DES-exposed MMSCs vs. DES-exposed DMCs and demonstrated that reprogramming of ERGs in MMSCs is cell-type specific. It is interesting to know the epigenetic difference between UFs and UFICs in future.

Although the loss of the *Tsc2* allele in the myometrium of Eker rats is rarely found in humans, the Eker rat model may be considered a phenocopy of the human situation in that *MED12* mutations occur in over 80% of patients with UFs. Given the fact that *MED12* mutations may appear due to a dysfunctional DNA repair system, one may consider that early-life exposure to EDCs hijacked epigenomic plasticity leading to hyper-responsiveness to estrogen hormones, eventually resulting in genomic abnormalities that may include *MED12* mutations. It is well documented that DNA damage is linked to hormone signaling pathways contributing to cancer development [52, 53]. Steroid hormones have been reported to be associated with the induction of genetic instability via multiple mechanisms. For instance, disruption of DNA repair pathways is closely associated with ER status. Several key initiators of the DNA damage response, including proteins, such as ataxia-telangiectasia mutated (ATM) and ATM- and Rad3-Related (ATR), are negatively regulated by ER in breast cancer. DNA repair deficiency has also been observed in prostate cancer, another hormone-dependent tumor [54]. We previously reported that developmental EDC exposure decreased DNA end-joining ability, increased DNA damage load, and impaired repair of DNA double-strand breaks in MMSCs [27], and vitamin D3 ameliorated DNA damage caused by early-life exposure to EDC [55]. Crosstalk between the DNA repair and abnormal hormone signaling may interfere with the DNA damage repair pathway, leading to decreased DNA repair capacity and triggering UF development. Like breast cancer, UF is a hormone-dependent disease in which estrogen signaling converges to suppress effective DNA repair. Importantly, this is the first report to demonstrate that many of the cellular components that participate in estrogen signaling are reprogrammed in MMSCs. By focusing specifically on these cells, this research offers a more comprehensive understanding of the underlying mechanisms driving UF pathogenesis, while circumventing the potential dilution effect that could arise from using heterogeneous uterine tissues [56].

**Fig. 9:**
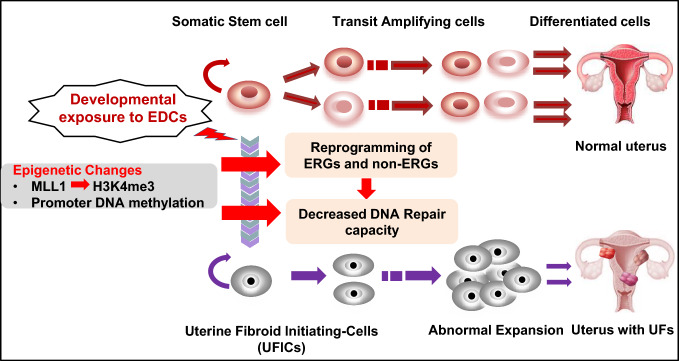
Model for developmental reprogramming of the epigenome in MMSCs. Environmental risk factors, including EDC exposure, disrupt the epigenome via histone modification and DNA methylation, thereby leading to the conversion of MMSCs into UF-initiating cells, and eventually giving rise to the formation of UFs

In this study, we also identified a paracrine effect of EDC-MMSCs/VEH-MMSCs on DMCs. The secretome from EDC-MMSCs enhanced DMC proliferation compared to that of VEH-MMSCs, concurrent with the activation of β-catenin signaling, one of the key pathways contributing to the growth and development of UFs. The paracrine effect of DMCs on a side population of human UF cells has been described previously [45]. Estrogen/progesterone selectively induces nuclear translocation of β-catenin, leading to the proliferation of a side population of UF cells. These data suggest that interaction between MMSCs and DMCs via a paracrine mechanism contributes to the pathogenesis of UFs, further demonstrating the important role of MMSCs in the development of UFs (Fig. 9).

Notably, our studies demonstrate that developmental exposure to EDC regulates multifaceted signaling pathways, possibly impairing uterine function, and leading to other uterine diseases. One such disease is preterm labor, which the progesterone receptor pathway can regulate. Changes in the ratio of progesterone receptor isoforms could impair the function of progesterone, and the premature onset of labor is associated with increased PR-A expression [57, 58]. Our data showed that *Pgr* is epigenetically reprogrammed (H3K4me3 enrichment), concomitant with increased expression of *Pgr* in EDC-exposed MMSCs. Moreover, labor requires increased expression of genes that mediate myometrial activation and optimal responsiveness to uterotonic agonists, such as stimulatory prostaglandins and oxytocin [57]. In addition to *Pgr*, we found that the oxytocin receptor (*Oxtr)* involved in labor was also H3K4me3-dependently reprogrammed in EDC-MMSCs.

Moreover, increased expression of prostaglandin-endoperoxide synthase 2* (Ptgs2),* another gene involved in labor, was observed in EDC-MMSCs compared to VEH-MMSCs. However, the H3K4me3 enrichment at *Ptgs2* was not observed, suggesting that an alternative mechanism is responsible for the altered expression of this gene. Additional studies are needed to determine whether *Ptgs2* expression is regulated by DNA methylation or other epigenetic mechanisms such as histone marks or miRNA. These abnormal reprogramming and increased expression of labor-related genes in MMSCs may promote preterm labor due to environmental exposures.

Several other EDCs, such as phthalates, have been shown to correlate with UF pathogenesis [11, 59]. Given the fact that each individual EDC and environmentally relevant EDC mixtures may impact the reproductive tissues differentially. Therefore, deep insight into the pathogenesis of UF induced by EDC exposures will help develop new therapies for the treatment and prevention of UF pathogenesis as precision medicine while limiting collateral damage and side effects.

This study used a pooled testing strategy combining MMSCs from multiple defined rats and testing them as a group for numerous cellular and molecular analyses. Since progenitor-type cells consist of a small percentage of populations in the myometrial and UF tissues [7, 19, 45], this strategy overcomes the challenges of overgrowing MMSCs, which may alter the characteristic of MMSCs. It is worth noting that investigating MMSC reprogramming in individual rats will further demonstrate the impact of developmental exposure to EDCs on the risk of UFs and help determine the EDC response for each animal using alternative technologies, such as single-cell multi-omics.

## Conclusions

We present compelling evidence demonstrating that xenoestrogens (EDCs) directly target and affect the epigenetics of MMSCs, which are the cell source for UFs. Through epigenomic profiling, we unveil new insights into the mechanisms that regulate the transcriptional landscape of MMSCs. Exposure to EDCs during early life, such as DES, causes significant changes in the expression patterns of multiple genes, including various estrogen-responsive genes (ERGs), mediated by histone and DNA methyltransferases in MMSCs. These alterations in gene expression modify the defining characteristics of MMSCs, leading to a "hyper-estrogenic" phenotype characterized by reduced DNA repair capacity and an elevated risk of hormone-dependent uterine diseases like UFs.

### Supplementary Information

Below is the link to the electronic supplementary material.

Supplementary file1 (XLSX 18 kb)

Supplementary file2 (XLSX 18 kb)

Additional File 3: Table S3, The methylation sites for ERGs (XLSX 19 kb)

Additional File 4: Fig. S1. ERG expression in EDC-MMSCs and VEH-MMSCs. a) Differential expression of ERGs in EDC-MMSCs compared to VEH-MMSCs identified by RNA-seq and bioinformatics analysis. B) Overlap of EDC-regulated genes in MMSCs with estrogen early and late response genes. Additional File 5: Fig. S2. Diagram of COMPASS complex and its pathway. Diagram showing the Compass complex linking to the active chromatin via H3K4me3 mark (left panel), and how MLL1 epigenetic pathways are activated by the protease Taspase 1 (right panel). Additional File 6: Fig. S3. Bisulfite NGS. Targeted bisulfite NGS: the quantitative methylation levels of 7 ERGs including Cxcl12, Ar, Bcl11b, CD9, Mpped2, Tgm2, and Pgr were measured by bisulfite NGS in VEH- and EDC-MMSCs. The Wilcoxon test was used to determine significant difference in the DNA methylation levels of eight ERGs covering 102 CpG sites around the promoter regions of these genes in EDC- and VEH-MMSCs. ***p<0.001. Additional File 7: Fig. S4. Integration of H3K4me3/DNA methylation near EDC-regulated genes. a) The Peaks/methylation near EDC-regulated genes. b) The overlap between H3K4me3 and RRBS (PDF 351 kb)

## Data Availability

The authors declare that omics data (RNA-seq, ChIP-seq and RRBS) supporting the findings of this study are available in GEO database with accession number GSE157503.

## Abbreviations

DEG: Differentially expressed gene
UF: Uterine fibroids
EDC: Endocrine-disrupting chemicals
DES: Diethylstilbestrol
MMSC: Myometrial stem cell
ERG: Estrogen-responsive genes
RRBS: Reduced Representation Bisulfite Sequencing
ERE: Estrogen-responsive element
MLL1: Mixed lineage leukemia protein-1
