# Supplementary material for: Developmental reprogramming of myometrial stem cells by endocrine disruptor linking to risk of uterine fibroids
Source: Cell Mol Life Sci. 2023 Aug 31;80(9):274. doi: 10.1007/s00018-023-04919-0 (PMC10471700; doi:10.1007/s00018-023-04919-0)
Supplement: Supplementary file 4 — Additional File 4: Fig. S1. ERG expression in EDC-MMSCs and VEH-MMSCs. a) Differential expression of ERGs in EDC-MMSCs compared to VEH-MMSCs identified by RNA-seq and bioinformatics analysis. B) Overlap of EDC-regulated genes in MMSCs with estrogen early and late response genes. Additional File 5: Fig. S2. Diagram of COMPASS complex and its pathway. Diagram showing the Compass complex linking to the active chromatin via H3K4me3 mark (left panel), and how MLL1 epigenetic pathways are activated by the protease Taspase 1 (right panel). Additional File 6: Fig. S3. Bisulfite NGS. Targeted bisulfite NGS: the quantitative methylation levels of 7 ERGs including Cxcl12, Ar, Bcl11b, CD9, Mpped2, Tgm2, and Pgr were measured by bisulfite NGS in VEH- and EDC-MMSCs. The Wilcoxon test was used to determine significant difference in the DNA methylation levels of eight ERGs covering 102 CpG sites around the promoter regions of these genes in EDC- and VEH-MMSCs. ***p<0.001. Additional File 7: Fig. S4. Integration of H3K4me3/DNA methylation near EDC-regulated genes. a) The Peaks/methylation near EDC-regulated genes. b) The overlap between H3K4me3 and RRBS (PDF 351 kb) [file 18_2023_4919_MOESM4_ESM.pdf]

Gene	DES over veh (log2FC)	E2 response, early	E2 response, late
Bcl11b	10.22	yes	
Reep1	8.00	yes	
Inhbb	7.91	yes	
Mdk	7.58		yes
Unc13b	7.28		yes
Cxcl12	7.25	yes	yes
Mapk13	6.84		yes
Pdlim3	5.94	yes	yes
Gfra1	5.89	yes	
Ar	5.59	yes	
Lrig1	5.47	yes	
Npy1r	5.01	yes	yes
Adcy9	4.72	yes	
Olfm1	4.23	yes	yes
Greb1	4.11	yes	
Homer2	3.81		yes
Tpd52l1	3.69	yes	yes
Tiam1	3.59	yes	yes
Krt18	3.39	yes	
Trim29	3.26		yes
Wisp2	3.13	yes	yes
Egr3	3.09	yes	yes
Tspan13	3.05		yes
Mpped2	2.98	yes	
Tnnc1	2.50		yes
Cd9	2.48		yes
Tgm2	2.41	yes	
Gale	2.25		yes
Areg	2.13	yes	yes
Mybl1	2.01	yes	
Tubb2b	2.01	yes	
Slc24a3	1.86	yes	yes
Scarb1	1.84	yes	yes
Sgk1	1.82		yes
Nrip1	1.63	yes	yes
Rps6ka2	1.61	yes	yes
Sybu	1.61	yes	
Pla2g16	1.56	yes	yes
Fdft1	1.52	yes	yes
Pgr	1.51	yes	yes
Zfp36	1.49		yes
Myof	1.47	yes	yes
B4galt1	1.44	yes	
Ccnd1	1.40	yes	yes
Rrp12	1.40	yes	
Hspb8	1.36	yes	yes
Klf4	1.31	yes	yes
Flnb	1.31	yes	yes
Abca3	1.28	yes	yes
Ugdh	1.21		yes
Sema3b	1.20	yes	yes
Lamc2	1.16		yes
Igf1r	1.14	yes	
Igfbp4	1.13	yes	yes
Endod1	1.13	yes	
Pdzk1	1.12	yes	yes
Fasn	1.10	yes	
Nbl1	1.08	yes	yes
Bcl2	1.02	yes	yes
St6galnac2	1.01		yes

Gene	DES over veh (log2FC)	E2 response, early	E2 response, late
Tbc1d30	-12.56	yes	
Plxnb1	-6.20		yes
Prkar2b	-4.06		yes
Scube2	-3.35		yes
Dhrs3	-2.41	yes	
Ablim1	-2.40	yes	
Top2a	-2.32		yes
Llgl2	-2.31		yes
Syngr1	-2.28	yes	
Scnn1a	-2.15	yes	yes
Frk	-2.08	yes	yes
Dlc1	-2.01	yes	
Cdc20	-1.77		yes
Kif20a	-1.72		yes
Gja1	-1.63	yes	
Kazn	-1.57	yes	
Svil	-1.55	yes	
Cpe	-1.49		yes
Itpk1	-1.48	yes	yes
Nab2	-1.47		yes
Tgif2	-1.35	yes	
Stil	-1.29		yes
Impa2	-1.16		yes
Sec14l2	-1.09	yes	
Stc2	-1.09	yes	
Dnajc12	-1.03		yes
Slc16a1	-1.02	yes	yes

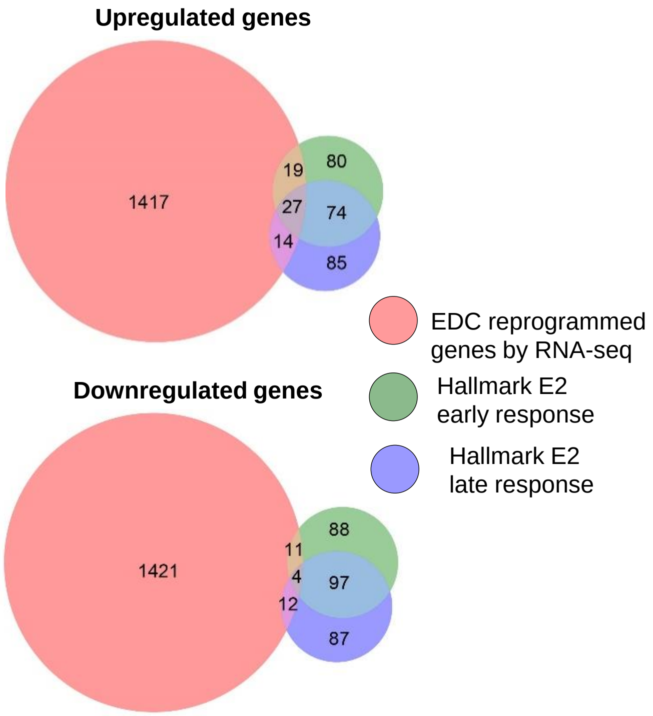

Additional file 4 Fig. S1

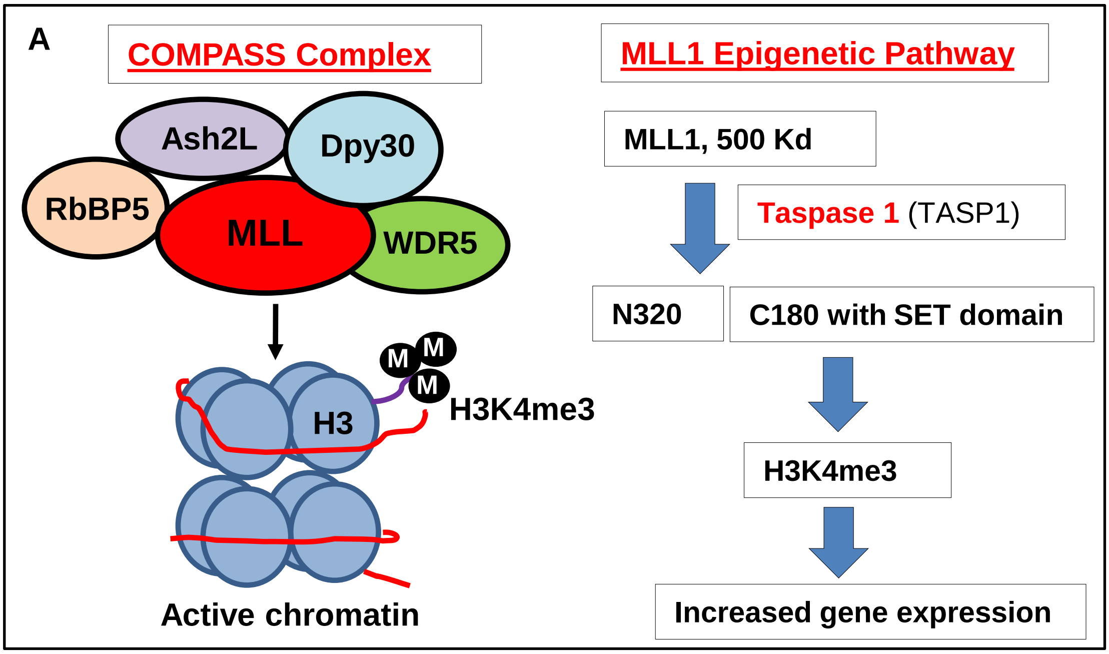

Additional file 5 Fig. S2

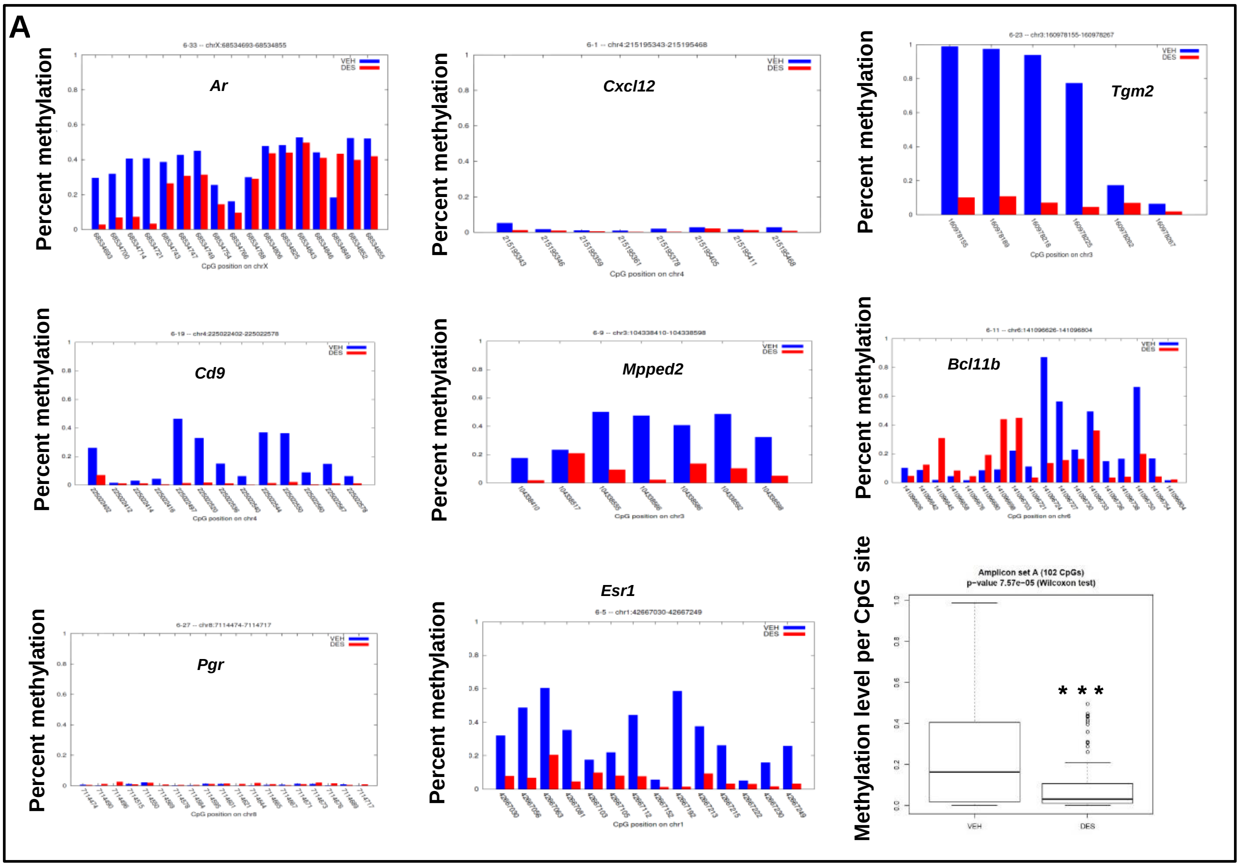

Additional file 6 Fig. S3

Peaks/methylation near DES-regulated genes

Strict definition: 4me3 or RBBS in one direction only, not mixed

Gene body +/– 10 kb

expected direction				opposite direction		
**DES-upregulated genes**	**1474**			**DES-upregulated genes**	**1474**	
RRBS down 10%	467	31.68%		RRBS up 10%	223	15.13%
RRBS down 33%	377	25.58%		RRBS up 33%	215	14.59%
RRBS down 50%	174	11.80%		RRBS up 50%	101	6.85%
4me3 up	694	47.08%		4me3 down	90	6.11%
**DES-down regulated genes**	**1448**			**DES-down regulated genes**	**1448**	
RRBS up 10%	173	11.95%		RRBS down 10%	462	31.91%
RRBS up 33%	147	10.15%		RRBS down 33%	391	27.00%
RRBS up 50%	80	5.52%		RRBS down 50%	180	12.43%
4me3 down	485	33.49%		4me3 up	157	10.84%

mixed direction				no mark		
**DES-upregulated genes**	**1474**			**DES-upregulated genes**	**1474**	
RRBS both 10%	327	22.18%		RRBS none 10%	456	30.94%
RRBS both 33%	111	7.53%		RRBS none 33%	769	52.17%
RRBS both 50%	14	0.95%		RRBS none 50%	1183	80.26%
4me3 both	97	6.58%		4me3 none	593	40.23%
**DES-down regulated genes**	**1448**			**DES-down regulated genes**	**1448**	
RRBS both 10%	331	22.86%		RRBS none 10%	482	33.29%
RRBS both 33%	123	8.49%		RRBS none 33%	787	54.35%
RRBS both 50%	20	1.38%		RRBS none 50%	1168	80.66%
4me3 both	138	9.53%		4me3 none	668	46.13%

overlap between 4me3 & RRBS		
**DES-upregulated genes**	**1474**	
4me3 up + RRBS down 10%	540	36.64%
4me3 up + RRBS down 33%	340	23.07%
4me3 up + RRBS down 50%	153	10.38%
**DES-down regulated genes**	**1448**	
4me3 down + RRBS up 10%	279	19.27%
4me3 down + RRBS up 33%	166	11.46%
4me3 down + RRBS up 50%	76	5.25%

Additional file 7 Fig. S4
